# Extent of implementation of the *Disaster Risk Reduction and Management Act (RA 10121)* in the schools of Cabadbaran City, Philippines

**DOI:** 10.4102/jamba.v18i1.1991

**Published:** 2026-01-13

**Authors:** Nathalie L. Daminar, Cora B. Cabonce

**Affiliations:** 1 Faculty of Teacher Education, College of Industrial Technology and Teacher Education, Caraga State University Cabadbaran Campus, Cabadbaran City, Philippines

**Keywords:** extent of implementation, disaster risk reduction, management, RA 10121, school-based disaster risk governance, student welfare policy

## Abstract

This study assessed the implementation of *Republic Act 10121*, the *Philippine Disaster Risk Reduction and Management Act*, within public schools in Cabadbaran City. The research addressed the effectiveness of programme execution across four thematic areas: prevention and mitigation, preparedness, response, and recovery. The study also examined the administrative capacity of school leaders responsible for disaster risk reduction and management (DRRM) functions. Using descriptive and correlational research designs, the study gathered data through survey analysis from 42 school administrators, including school heads and DRRM coordinators. Results indicated consistent implementation ratings across all thematic areas, with preparedness receiving the highest rating. School administrators demonstrated capacity in institutional planning, resource management, and community engagement. The analysis confirmed a strong positive relationship between programme implementation and administrative capacity, indicating that leadership competence supported effective disaster management. Gaps persisted in infrastructure resilience, financing, and sustained risk reduction.

**Contribution:**

The study affirmed the need for policy integration and continued capacity development to reinforce school-based disaster risk governance.

## Introduction

The Philippines is consistently ranked among the most hazard-exposed countries in the world because of its geographical position along the Pacific Ring of Fire and the Western Pacific typhoon belt, making it highly vulnerable to typhoons, floods, earthquakes, and landslides that threaten human lives, infrastructure, and institutions. This frequent exposure brings out the urgency of strengthening disaster risk reduction and management (DRRM) and climate change adaptation (CCA) mechanisms (Ner et al. 2022; United Nations Office for Disaster Risk Reduction [UNDRR] 2019).

*Republic Act No. 10121*, known as the *Philippine Disaster Risk Reduction and Management Act of 2010*, offers a policy and legal basis for a multisectoral, progressive, and proactive DRRM framework. The Act restructured the National Disaster Coordinating Council into the National Disaster Risk Reduction and Management Council (NDRRMC) and decentralised the responsibilities to the local government units (LGUs), civil society and local institutions (NDRRMC 2011). It specifically requires the incorporation of DRRM into development planning, education, infrastructure practices, and community-based programmes. Disaster risk reduction and management is operationalised in the law as four thematic areas, namely, (1) prevention and mitigation, (2) preparedness, (3) response, and (4) recovery and rehabilitation (Castillo, Deleon III & Delgado 2024).

Within this framework, the Department of Education-institutionalised DRRM became institutionalised in schools with Department of Education (DepEd) Orders 55 and 50 series of 2007 and 2011, which require schools to develop contingency plans, carry out capacity-building exercises, deploy structural and non-structural mitigation actions, and support community resilience (Dela Cruz & Ormilla 2022). The schools are not only the learning centres but also the emergency evacuation centres and other safety points in society in case of a disaster. Nevertheless, empirical evaluations indicate that there are consistent gaps in the four DRRM thematic areas. In terms of prevention and mitigation, studies indicated that schools are faced with the problems of retrofitting older infrastructure, land-use compliance, and hazard mapping because of financial and technical limitations (Baluran 2023; Tizon & Comighud 2020). Regarding preparedness, earthquake and fire drills are highly practised, but their quality, frequency, and the rate of participation differ significantly, with the poor schools in rural areas trailing in this area (Pandapatan 2024; Viado 2023). Regarding response mechanisms, it has been shown that there are few emergency resources in the form of first aid kits, functional communication systems, and updated incident command structures (Cortejo, Ignes & Bangonon 2024). The processes of recovery and rehabilitation are mostly reactive as schools have reported delays in damage assessment, psychosocial care, and classroom rehabilitation following a disaster because of administrative bottlenecks and insufficient funding (Gaudiel 2023; Lai et al. 2016). These indicate skewed adherence to the requirements of *RA 10121* on school-based DRRM.

Despite these national directives and institutional mandates, systematic assessments at the city level remain scarce, particularly in Cabadbaran City in the Caraga Region, which is highly exposed to hydrometeorological and geophysical hazards such as flooding, landslides, and earthquakes associated with active fault systems (Marfito, Llamas & Aurelio 2022; Santillan, Makinano-Santillan & Cutamora 2016). While the city maintains a functional DRRM Office that coordinates with schools, no empirical investigation has yet evaluated the extent of compliance of public schools with *RA 10121* across the four thematic areas, thereby constraining evidence-based policymaking and limiting the ability of school administrators and local governments to design hazard-specific interventions. This study addresses this gap by assessing the extent of DRRM implementation in public schools in Cabadbaran City, examining the capability of school administrators in performing DRRM-related functions, and analysing the relationship between implementation and administrative capacity. The findings are expected to provide empirical evidence that can support policy formulation, guide resource allocation, and strengthen institutional resilience at both the local and national levels by advancing the discourse on the intersection of administrative capacity and DRRM implementation in the education sector.

### Objectives of the study

This study seeks to evaluate the status of DRRM implementation in public schools in Cabadbaran City within the framework of *Republic Act No. 10121*. It aims to determine the extent to which the four thematic areas of DRRM, namely, prevention and mitigation, preparedness, response, and recovery, are integrated into school operations, programmes, and practices. The study also intends to assess the capability of school administrators in carrying out their DRRM-related responsibilities, recognising their role as primary agents in institutionalising risk reduction and preparedness initiatives at the school level. In addition, it seeks to examine the relationship between the degree of DRRM implementation and the administrative capability of school heads, with the objective of generating empirical evidence that can clarify whether institutional capacity influences the effectiveness of policy execution. By formulating these objectives, the research positions itself to provide a systematic evaluation of localised DRRM practices and to contribute actionable insights for enhancing disaster resilience in the education sector.

### Conceptual framework of the study

**FIGURE 1:**
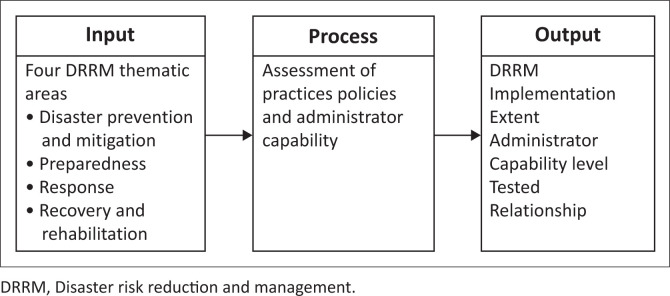
Conceptual framework (Input–Process–Output model).

This study anchored its framework on *Republic Act No. 10121* and the National Disaster Risk Reduction and Management Plan 2011–2028 (NDRRMC 2011), which institutionalised DRRM as a shared responsibility among government agencies, schools, and communities. Within this framework, schools are positioned as critical sites for disaster prevention, preparedness, response, and recovery, serving both protective and educational functions. The four thematic areas of DRRM, namely, prevention and mitigation, preparedness, response, and recovery and rehabilitation, were conceptualised as the independent variables of the study, while the capability level of school administrators was treated as a moderating variable. The relationship between DRRM implementation and administrative capability reflected both compliance with statutory mandates and the institutional readiness of schools to manage hazards. To illustrate this relationship, the study adopted the Input–Process–Output (IPO) model (Figure 1), where the input comprised the four thematic areas, the process entailed the assessment of practices, policies, and administrator capability, and the output generated measures of DRRM implementation, administrator capability level, and the tested relationship between the two. This paradigm linked national statutory requirements with localised institutional actions and compliance outcomes, thereby providing a structured guide for data gathering, analysis, and formulation of recommendations.

## Literature review

Disaster risk reduction and management in the Philippines is influenced by international and local policies. At the global scale, the Sendai Framework of Disaster Risk Reduction (2015–2030) focuses on the four priority areas, which include: (1) the understanding of disaster risk, (2) enhancing disaster risk governance, (3) investing in disaster reduction to build resilience, and (4) strengthening a disaster preparedness to respond effectively and build back better through recovery, rehabilitation, and reconstruction (UNDRR 2019).

Although these themes are a complete roadmap, the school level has encountered the difficulties of low-level risk awareness, the lack of integration of DRR concepts in the curriculum, resource deficit, and ineffective monitoring systems (Abregana 2025; Wisner 2020). Such gaps represent the truth that policies, despite their well-formulation, can be challenged to be converted into uniform school-based practices, particularly in communities with limited resources and those where hazards are evident.

The *Republic Act No. 10121* or the *Philippine Disaster Risk Reduction and Management Act of 2010* institutionalised a comprehensive DRRM system at the national level. The Act has formed the NDRRMC and requires DRRM mainstreaming in development planning, which also includes the education sector (Congress of the Philippines 2010). It states that schools, via their DRRM coordinators and administrative heads, are to embrace certain measures within the four thematic areas, which are prevention and mitigation, preparedness, response, and rehabilitation and recovery. This is a legal framework that will inform the instruments and evaluations design, including this study. Nevertheless, even with the provisions of the law, researchers point out such barriers to its implementation as budget restrictions, insufficient training of school staff, and uneven local government assistance (Acierto, Robas & Monte 2023; Luminarias & Liquido 2025). Thus, the investigation of the ways schools apply *Republic Act 10121* in particular contexts of hazards is one of the urgent concerns.

The summary of the selected Philippine studies on the school-based DRRM implementation is given in Table 1. Baluran (2023) demonstrated that schools proactively implemented DRRM protocols that matched the four thematic areas but were different in the extent of preparedness based on the differences in resources and contextual vulnerabilities. On the same note, Viado (2023) has discovered that administrators and teachers were familiar with the DRRM policies, but there were imbalanced practices in schools due to financial constraints and irregular support by local governments. As highlighted by Pandapatan (2024), the schools followed the required drills and information dissemination during COVID-19, but the sustainability of mitigation and recovery efforts was compromised due to the limitations of school budgets, which is why schools need external assistance.

**TABLE 1:** Related studies on the status of the schools’ disaster risk reduction and management implementation.

Author-year	Location	Respondents	Focus	Key findings
Pandapatan (2024)	Iligan City	81 teachers	DRRM execution during COVID-19	Campuses complied with the four thematic areas and national directives.
Baluran (2023)	Polomolok, South Cotabato	40 district coordinators	Implementation levels across three districts	South and West rated high in prevention mitigation; preparedness varied (South high, East moderate, West low); response high across districts; recovery high in West and East, intermediate in South
Viado (2023)	Zambales	6 campuses	Readiness of students and staff	Teaching staff moderate on planning and warning system tasks; students moderate on warning system tasks; other domains showed readiness.
Dela Cruz and Ormilla (2022)	Alfonso Lista District, Ifugao	36 school heads + DRRM coordinators	Link between preparedness and implementation	Secure learning facilities in place; DRRM integrated into curriculum; staff understanding correlated with adoption levels.
Tizon and Cominghod (2020)	Bayawan City, Negros Oriental	96 school heads	Evaluation of implementation	DRRM well-implemented; administrator capability correlated with implementation status.
Regis (2020)	Region VIII (Eastern Visayas)	469 DRRM team members	Extent of thematic-area execution and capability	Relief and rehabilitation scored moderate; other areas scored high; implementation differed with training volume and education level.

Note: Please see the full reference list of this article, Daminar, N.L. & Cabonce C.B., 2026, ‘Extent of implementation of the Disaster Risk Reduction and Management Act (RA 10121) in the schools of Cabadbaran City, Philippines’, *Jàmbá: Journal of Disaster Risk Studies* 18(1), a1991. https://doi.org/10.4102/jamba.v18i1.1991, for more information.

COVID-19, coronavirus disease; DRRM, disaster risk reduction and management.

Other researchers have also pointed out the role of administrators and organisational capacity towards the formation of outcomes. Tizon and Comighud (2020) noted that the capability among administrators had a very strong relationship with the degree of implementation, whereas Gaudiel (2023) also reported that leadership competence was the determining factor in the quality of preparedness and response efforts. By this time, Cortejo et al. (2024) identified an imbalance between developing response mechanisms that were highly developed and recovery and long-term resilience, which were also poorly developed. Such results are consistent with previous studies conducted by Regis (2020), which reported that recovery programme implementation was less efficient than preventive and preparedness activities, and that disparities depended on the training exposure and education levels of members of DRRM teams.

Taken together, these studies demonstrate that while DRRM activities are present in many schools, implementation varies significantly depending on resources, administrative capability, and local hazard contexts. None of the existing research, however, has focused specifically on Cabadbaran City. The city represents a critical site for investigation due to its high exposure to hydrometeorological and geophysical hazards. Its river basin systems and mountainous terrain are the reasons that make it highly vulnerable to flooding and landslides caused by rain (Santillan et al. 2016). It is also close to the portions of the Philippine Fault Zone, which exposes it to seismic activities (Marfito et al. 2022). Although DRRM-related studies have been conducted on these Philippine areas, including Iligan City, South Cotabato, and Ifugao, because of significant disaster incidents, no scholarly work has been done on Cabadbaran despite the same or even greater exposure to hazards (Baluran 2023; Dela Cruz & Ormilla 2022; Pandapatan 2024). This lack of empirical assessment is significant because the Caraga Region ranks among the top regions in terms of disaster frequency and vulnerability, yet remains underrepresented in DRRM literature (Varela, Apdohan & Balanay 2022). All in all, this gap highlights the importance of the present study, which aims to provide a systematic assessment of DRRM implementation in Cabadbaran’s public schools and to analyse the role of administrator capability in achieving compliance with *Republic Act No. 10121*.

## Research methods and design

This study employed a quantitative descriptive survey design to assess the status of DRRM implementation and the capability levels of school administrators in Cabadbaran City. The approach was selected to provide a structured analysis of existing practices within schools under the Department of Education. The study focused on describing current conditions without manipulating variables, which is appropriate for evaluating policy execution and administrative preparedness.

### Data collection

Data were collected through a structured survey questionnaire administered to 42 school administrators, specifically the School Head and the DRRM Coordinator from 21 schools. These respondents were selected through a purposive sampling to ensure that they had direct, documented involvement in school-based DRRM planning and implementation. The questionnaire was divided into two sections. The first section measured the DRRM implementation status, which was developed directly from *RA 10121* and the NDRRMC Plan 2012–2028 (NDRRMC 2011). The second section assessed administrator capability and was adapted from Tizon and Comighud (2020), drawing on the principles of the Hyogo Framework for Action (HFA). To identify the validity and clarity of the survey tool, the panel of three subject matter experts reviewed the questionnaire with regard to its content relevance, clarity, and theoretical consistency. Additionally, to minimise the risk of bias in the survey response, researchers strictly adhered to the confidentiality of the respondents and had two key informants per school so as to have a triangulated and balanced view of the school’s performance. The respondents measured the implementation of DRRM in schools on a 5-point Likert scale and gathered the data in person, with prior coordination and consent.

### Data analysis

Data were analysed using descriptive and inferential statistics. The mean and standard deviation (s.d.) were computed to describe the extent of DRRM implementation and the level of administrator capabilities. Interpretation of mean scores for DRRM implementation was based on a scale adapted from (Gaudiel 2023), while capability levels were interpreted according to the categories provided in the study instrument. To determine the relationship between DRRM implementation and administrator capability levels, the study employed Pearson’s correlation coefficient. A *t*-test was also used to identify significant differences in DRRM implementation and capability levels based on selected respondent characteristics.

### Ethical considerations

Ethical clearance to conduct this study was obtained from the Caraga State University Ethics Review Committee (No. CU-ERC-2025-014).

## Results

Findings demonstrate that the implementation of DRRM in Cabadbaran City’s public schools is consistently strong across all thematic areas, with all domains rated ‘Good Implementation’. Among these, disaster preparedness emerged as the most robustly implemented area, reflecting institutional commitment to proactive planning and risk awareness. The administrators also demonstrated a commendable level of competence across all capability indicators.

### Extent of disaster risk reduction and management implementation

Table 2 presents the extent of DRRM implementation in Cabadbaran City public schools across the four thematic areas of prevention and mitigation, preparedness, response, and recovery and rehabilitation. The overall mean score across all areas was 3.822 with a standard deviation (s.d.) of 0.934, which is interpreted as ‘Good’.

**TABLE 2:** Extent of disaster risk reduction and management implementation in Cabadbaran City public schools.

Thematic area	Overall mean	s.d.	Interpretation
Prevention and mitigation	3.82	0.92	Good
Preparedness	3.87	1.00	Good
Response	3.73	0.87	Good
Recovery and rehabilitation	3.65	1.14	Good
Grand mean	3.82	0.93	Good

s.d., standard deviation.

Among the four thematic areas, preparedness obtained the highest overall mean (*M* = 3.872, s.d. = 0.996), followed closely by prevention and mitigation (*M* = 3.822, s.d. = 0.922). Response was slightly lower with an overall mean of 3.727 (s.d. = 0.875), while recovery and rehabilitation registered the lowest score (*M* = 3.651, s.d. = 1.140). Despite variation across the thematic areas, all dimensions were consistently rated as good, indicating that schools are able to meet the minimum requirements of DRRM implementation. The results suggest that Cabadbaran City schools have institutionalised DRRM practices in line with *Republic Act No. 10121*. Strong performance in preparedness and prevention reflects the emphasis placed on drills, training, awareness campaigns, and policy integration in school systems. However, the relatively lower scores in response and recovery indicate that while schools can initiate immediate relief and protective measures, there are challenges in sustaining long-term rehabilitation programmes and in ensuring comprehensive post-disaster support. The variability in scores also implies that institutional priorities may be more focused on readiness rather than on post-crisis interventions, which could limit the overall resilience of schools in the face of recurring hazards.

These findings align with the observations of Baluran (2023), who noted that schools in the Philippines generally integrate DRRM activities but show variation in depth depending on resources and capacity. Similarly, Viado (2023) highlighted that preparedness is widely practised but that institutional responses remain constrained by financing limitations. Tizon and Comighud (2020) also reported that while preventive measures are introduced, their integration into school programmes is uneven. Moreover, Cortejo et al. (2024) observed that schools tend to emphasise immediate response mechanisms while giving less attention to recovery. The low rating for rehabilitation and recovery in this study further echoes the concerns raised by Gaudiel (2023) and Pandapatan (2024), who found that resource allocation and administrative capacity are critical challenges in sustaining DRRM efforts at the school level.

Table 3 presents the detailed indicators of DRRM implementation in Cabadbaran City public schools, disaggregated by thematic area. The findings show consistent ratings of ‘Good’ across all indicators, with overall means ranging from 3.651 for recovery and rehabilitation to 3.874 for preparedness. The results imply that Cabadbaran City schools are relatively strong in implementing environmental management, capacity-building, and damage assessment practices, which suggests a proactive orientation towards risk preparedness and post-disaster assessment. However, the consistently lower ratings in financial readiness, stakeholder coordination, temporary shelter provision, and psychosocial well-being highlight structural and institutional gaps. These findings suggest that while schools have made progress in policy integration and basic preparedness measures, they face challenges in sustaining more complex interventions that require cross-sectoral collaboration and specialised resources. These results align with the observations of Baluran (2023), who emphasised the uneven depth of DRRM implementation across schools depending on available resources. Viado (2023) also noted that preparedness activities such as training and awareness campaigns are often prioritised, while resource-intensive aspects of response and recovery are more difficult to sustain. The findings also echo Cortejo et al. (2024), who observed that schools often excel in immediate response activities but lag in rehabilitation and recovery planning. Similarly, Gaudiel (2023) emphasised the role of administrators in ensuring implementation, while Pandapatan (2024) identified financing as a persistent constraint. Taken together, the present results confirm existing literature while providing localised evidence from Cabadbaran City, particularly emphasising psychosocial recovery and stakeholder coordination as underdeveloped areas.

**TABLE 3:** Detailed indicators of disaster risk reduction and management implementations.

Thematic area	Indicators	Mean	s.d.	Interpretation
Prevention and mitigation	DRRM and CCA in policies and budgets	3.91	0.93	Good
	Environmental management	4.03	0.82	Good
	Resilient infrastructure	3.77	0.96	Good
	Scientific risk assessment	3.91	0.91	Good
	Risk financing and insurance	3.62	1.13	Good
	Early warning systems	3.69	0.78	Good
	Overall	3.82	0.92	Good
Preparedness	Community awareness	3.91	0.93	Good
	Skills and capacity	4.03	0.82	Good
	Infrastructure resiliency	3.77	0.96	Good
	Policy and planning	3.91	0.91	Good
	Stakeholder coordination	3.62	1.13	Good
	Overall	3.87	1.00	Good
Response	Relief operations	3.91	0.93	Good
	Damage and needs assessment	4.03	0.82	Good
	Search, rescue, and retrieval capacity	3.77	0.96	Good
	Evacuation procedures	3.91	0.91	Good
	Temporary shelters	3.62	1.13	Good
	Basic services	3.82	0.92	Good
	Psychosocial support	3.91	0.93	Good
	Early recovery	4.03	0.82	Good
	Overall	3.79	0.87	Good
Recovery and rehabilitation	Damage and loss assessment	3.91	0.93	Good
	Economic recovery	4.03	0.82	Good
	DRRM in human settlements	3.77	0.96	Good
	Resilient infrastructure	3.91	0.91	Good
	Psychological well-being	3.62	1.13	Good
	Overall	3.65	1.14	Good

DDRM, disaster risk reduction management; CCA, climate change adaptation; s.d., standard deviation.

### Capabilities of school administrators

Table 4 presents the level of administrator capacities in Cabadbaran City public schools, measured across five indicators. The overall results indicate that administrators were assessed as ‘Capable’ in all dimensions, with mean scores ranging from 3.697 to 3.765. Among the five indicators, capacities and mechanisms obtained the highest mean score (*M* = 3.765, s.d. = 0.867), suggesting that administrators demonstrate relatively strong institutional mechanisms for implementing DRRM. This was followed closely by knowledge and education (*M* = 3.735, s.d. = 0.866) and policies and procedures (*M* = 3.722, s.d. = 0.900), which reflect administrators’ familiarity with DRRM principles and compliance with institutional guidelines. Human resources (*M* = 3.702, s.d. = 0.856) and material facilities (*M* = 3.697, s.d. = 0.934) received the lowest ratings, indicating limitations in staffing and physical resources to fully support DRRM programmes.

**TABLE 4:** Level of administrator capacities.

Capacity indicators	Mean	s.d.	Interpretation
Human resources	3.70	0.86	Capable
Material facilities	3.70	0.93	Capable
Knowledge and education	3.73	0.87	Capable
Policies and procedures	3.72	0.90	Capable
Capacities and mechanisms	3.76	0.87	Capable

s.d., standard deviation.

The findings suggest that while administrators possess adequate knowledge, procedures, and institutional mechanisms to perform DRRM responsibilities, the capacity to mobilise human and material resources remains a challenge. This highlights an imbalance between technical knowledge and the logistical or operational support needed to implement DRRM effectively. The results imply that administrator capability is strongest in planning and organisational aspects but is constrained in terms of resources, which may hinder the full translation of policy into practice at the school level.

These findings are consistent with Gaudiel (2023), who emphasised that administrator leadership and competence strongly influence the success of school-level DRRM implementation. However, as Pandapatan (2024) pointed out, resource limitations, such as insufficient facilities and financing, continue to undermine the ability of schools to sustain programmes, a challenge also evident in the lower scores for human resources and material facilities in this study. Similarly, Viado (2023) highlighted that institutional preparedness often relies heavily on leadership capacity but is weakened when resource support is lacking. The present findings therefore reinforce the view that administrator capability is multidimensional, requiring not only knowledge and organisational skills but also sufficient human and material inputs to ensure resilient school systems.

### Correlation between disaster risk reduction and management implementation and capabilities

The Pearson correlation coefficient showed a strong, significant relationship between the DRRM implementation status and the level of administrator capability.

A key result of the study is the strong positive correlation (*r* = 0.879, *p* < 0.01) between the extent of DRRM implementation and the capability of school administrators. This finding affirms that effective programme execution is closely tied to the skill, preparedness, and institutional support available to administrators. The result supports theoretical and empirical frameworks in disaster governance that emphasise the integration of systems, leadership, and preparedness (NDRRMC 2011; Tizon & Comighud 2020). The result indicates that higher levels of administrator capacity are strongly associated with higher levels of DRRM implementation. This suggests that as school administrators demonstrate greater competence in human resource management, material facility utilisation, policy enforcement, and institutional mechanisms, the effectiveness of DRRM programmes also improves. The significance of the *p*-value confirms that this relationship is unlikely to have occurred by chance, bringing out the reliability of the observed correlation.

The findings imply that administrator capacity is not merely supportive but a determining factor in the success of DRRM implementation at the school level. Administrators with stronger leadership, organisational ability, and resource management skills are more effective in institutionalising DRRM, while limitations in these areas can constrain compliance with *Republic Act No. 10121*. The strong positive correlation reinforces the view that the role of administrators is pivotal in transforming policy frameworks into actionable practices that ensure preparedness, response, and recovery within schools. This finding is consistent with Gaudiel (2023), who emphasised that school administrators serve as the linchpin for effective DRRM implementation, with their leadership directly influencing outcomes. Similarly, Baluran (2023) and Viado (2023) highlighted that the depth of DRRM adoption varies across schools depending on the competence of administrators and the support systems available to them. Pandapatan (2024) also noted that administrative capability and access to resources are critical for sustaining DRRM programmes, particularly in under-resourced settings. The strong correlation observed in this study confirms these earlier findings, while providing empirical evidence from Cabadbaran City that administrator capacity is a decisive factor in ensuring effective disaster resilience in schools.

## Discussion

The results of this study provide empirical evidence on the extent of DRRM implementation in Cabadbaran City public schools and the role of administrator capacity in shaping these outcomes. The overall findings showed that implementation across the four thematic areas of prevention and mitigation, preparedness, response, and recovery and rehabilitation was consistently rated as ‘Good’ (*M* = 3.822, s.d. = 0.922), indicating institutional compliance with *Republic Act No. 10121* but revealing critical gaps that prevent schools from reaching higher levels of resilience.

A closer examination of the thematic areas revealed strengths in preparedness (*M* = 3.872, s.d. = 0.996) and prevention (*M* = 3.822, s.d. = 0.922), which were the two highest-rated areas (see Table 2). Schools reported high performance in environmental management, skills development, and damage assessment with a mean of 4.031 (s.d. = 0.822 as shown in Table 3), suggesting that programmes requiring routine training and awareness are more easily institutionalised. These results resonate with the findings of Baluran (2023) and Viado (2023), who documented the widespread practice of preparedness activities such as drills, awareness campaigns, and training in Philippine schools. The prioritisation of preparedness reflects a tendency of educational institutions to focus on readily implementable interventions that can be carried out within existing organisational structures.

However, the study also found relatively lower ratings in response (*M* = 3.727, s.d. = 0.875) and recovery and rehabilitation (M = 3.651, s.d. = 1.140), the two lowest-rated thematic areas (see Table 2), particularly in temporary shelter provision, stakeholder coordination, and psychosocial well-being with a mean of 3.625 (s.d. = 1.129 as shown in Table 3). These findings mirror the conclusions of Cortejo et al. (2024), who observed that schools are effective in executing immediate response mechanisms but are less capable of sustaining long-term rehabilitation measures. Similarly, Pandapatan (2024) emphasised that financial and resource constraints limit schools’ ability to maintain programmes beyond the initial stages of disaster response. The consistently lower scores in recovery and rehabilitation in this study underscore a structural gap in DRRM implementation: while schools can respond to immediate needs, they struggle with interventions that demand long-term planning, resource mobilisation, and interagency collaboration.

The role of administrators emerged as a critical determinant of these outcomes. The results showed that administrators were rated as ‘Capable’ across all dimensions, with their strongest capacities found in institutional mechanisms (*M* = 3.765, s.d. = 0.867), knowledge and education (*M* = 3.735, s.d. = 866), and policies and procedures (*M* = 3.722, s.d. = 0.900). However, human resources (*M* = 3.702, s.d. = 0.856) and material facilities (*M* = 3.697, s.d. = 0.934) received the lowest ratings (see Table 4), indicating limitations in operational support. This confirms the argument of Gaudiel (2023) that administrator leadership and competence are central to the effectiveness of school-based DRRM, but also highlights the systemic resource constraints noted by Pandapatan (2024). Administrators thus embody both the strength and the vulnerability of the school DRRM system: they possess the knowledge and institutional tools but are constrained by material deficits that limit the full execution of programmes.

Most significantly, the correlation analysis revealed a strong and statistically significant positive relationship (*r* = 0.879, *p* < 0.01) between administrator capacity and DRRM implementation (see Table 5). This finding confirms that administrative capability is not simply a supportive element but a decisive factor in determining the success of DRRM. It aligns with the conceptual framework of this study, which posits that the four thematic areas of DRRM serve as independent variables while administrator capacity functions as a moderating factor. The strength of this correlation provides empirical support for the proposition that schools with more capable administrators are better positioned to translate national policy into localised action. This result validates the insights of Baluran (2023), Viado (2023), and Gaudiel (2023), who highlighted administrative leadership as a key driver of implementation, while also reinforcing the argument of Pandapatan (2024) that resource availability remains intertwined with capacity.

**TABLE 5:** Correlation between disaster risk reduction and management implementation and capability.

Variable pair	*r*	*p*-value	Interpretation
Implementation x capacity	0.88	0.001	Significant, strong positive

Overall, the discussion features three critical implications. Firstly, the emphasis on preparedness and prevention indicates partial alignment with the statutory framework, but also exposes the need to strengthen the less-developed dimensions of response and recovery. Secondly, administrator capacity is proven to be both an asset and a bottleneck, depending on whether it is supported by adequate resources. Thirdly, the strong correlation between capacity and implementation suggests that policy interventions aimed at building administrator competence and providing material support will likely yield significant improvements in DRRM outcomes.

Situating these findings within the broader literature and the statutory framework of *Republic Act No. 10121*, this study affirms that DRRM in schools is both a governance and a capacity-building challenge. Schools can adopt policies and conduct preparedness activities, but their resilience depends on the ability of administrators to mobilise resources, sustain programmes, and coordinate with stakeholders. These conclusions reinforce the imperative of investing in both human and institutional capacity if DRRM in the education sector is to move beyond compliance and achieve comprehensive disaster resilience.

## Conclusion

This study assessed the implementation of *Republic Act 10121* in public schools in Cabadbaran City. The assessment addressed the four DRRM areas and the administrative capacities of school leaders. Results showed that schools maintained consistent levels of implementation across prevention and mitigation, preparedness, response, and recovery. Preparedness received the highest rating. School administrators demonstrated capacity across institutional dimensions. Human and organisational structures for disaster management operated as intended. The connection between programme execution and administrative capacity confirmed that leadership competence aligned with programme effectiveness. Schools demonstrated readiness and resilience. Gaps persisted in physical infrastructure, financing, and sustained risk reduction capacity. Findings affirmed the need to strengthen school-based disaster management through policy, governance, and institutional development. Results supported the integration of preparedness measures into long-term education planning to secure school communities.

### Implications for policy and practice

The results offer several practical implications. Firstly, maintaining and strengthening preparedness efforts, especially those related to community engagement and policy coordination, can serve as a model for scaling success across other DRRM areas. Secondly, strategic focus is needed in recovery and rehabilitation, particularly in post-disaster infrastructure and financing access. Thirdly, continuing to invest in administrator capability is likely to yield system-wide benefits in DRRM execution and sustainability. For policymakers, these findings support the value of integrating DRRM indicators into school performance monitoring systems. For local governments and DepEd divisions, they suggest a priority focus on logistics, DRR capacity, and post-disaster assessment tools.
